# Emergency surgery for gastrointestinal cancer: A nationwide study in Japan based on the National Clinical Database

**DOI:** 10.1002/ags3.12353

**Published:** 2020-06-21

**Authors:** Nobuaki Hoshino, Hideki Endo, Koya Hida, Nao Ichihara, Yoshimitsu Takahashi, Hiroshi Hasegawa, Toshimoto Kimura, Yuko Kitagawa, Yoshihiro Kakeji, Hiroaki Miyata, Takeo Nakayama, Yoshiharu Sakai

**Affiliations:** ^1^ Department of Surgery Kyoto University Graduate School of Medicine Kyoto Japan; ^2^ Department of Health Informatics Kyoto University School of Medicine and Public Health Kyoto Japan; ^3^ Department of Healthcare Quality Assessment Tokyo University Graduate School of Medicine Tokyo Japan; ^4^ Department of Surgery Kobe University Graduate School of Medicine Kobe Japan; ^5^ Department of Surgery Iwate Medical University School of Medicine Iwate Japan; ^6^ The Japanese Society of Gastroenterological Surgery Tokyo Japan; ^7^ Database Committee The Japanese Society of Gastroenterological Surgery Tokyo Japan

**Keywords:** emergency surgery, gastrointestinal neoplasms, morbidity, mortality

## Abstract

**Background:**

Emergency gastrointestinal surgery, although rare, is known for its high mortality and morbidity. However, the risks of emergency surgery for gastrointestinal cancer have not been investigated in depth. This study aimed to investigate the impact of emergency surgery on mortality and morbidity in patients with gastrointestinal cancers and to identify associated risk factors.

**Methods:**

We extracted data from the National Clinical Database, a nationwide surgery registration system in Japan, for patients with gastrointestinal cancer who underwent esophageal resection, total gastrectomy, distal gastrectomy, right hemicolectomy, or low anterior resection between 2012 and 2017. The impacts of emergency surgery on 30‐day mortality and incidence of overall postoperative complications were compared with those of non‐emergency surgery. Risk factors for mortality and overall postoperative complications were then sought in patients who underwent emergency surgery.

**Results:**

Thirty‐day mortality and incidence of overall postoperative complications were significantly higher in emergency surgeries for gastric, colon, and rectal cancers than in non‐emergency surgeries (odds ratios 4.86‐6.98 and 1.68‐2.18, respectively; all *P *< .001). Various risk factors were identified in the group that underwent emergency surgery, including preoperative sepsis and lower body mass index. Some of the risk factors were common to all types of surgery and others were specific to a certain type of surgery.

**Conclusion:**

The actual risk of emergency surgery and the risk factors for overall postoperative complications in emergency cases are shown to serve as a reference for postoperative management. Emergency surgery had an additional burden on patients depending on the type of surgery.

## Graphical abstract

This article shows the impact of emergency surgery on mortality and morbidity in patients with gastrointestinal cancers and associated risk factors by using a nationwide registry in Japan. Our findings are expected to serve as a reference for postoperative management.

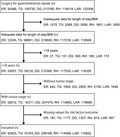

## 1 INTRODUCTION

Emergency surgery for gastrointestinal cancer is known to have high mortality and a high incidence of postoperative complications.^1^ Postoperative mortality is a worldwide problem and was reported to be the third leading cause of death globally in 2016.^2^ Emergency gastrointestinal surgery is usually performed only when patients have critical sudden‐onset symptoms, such as intestinal bleeding, intestinal stenosis, and peritonitis as a result of gastrointestinal perforation.^3^, ^4^, ^5^, ^6^, ^7^, ^8^, ^9^, ^10^, ^11^, ^12^, ^13^ Therefore, patients who require emergency surgery are in a worse condition than those who undergo non‐emergency surgery. However, with the exception of some procedures, such as stent placement for colonic stenosis, emergency surgery is likely to be unavoidable despite the poor condition of these patients.^14^

Although it is well known that patients undergoing emergency surgery have higher mortality and morbidity than those undergoing non‐emergency surgery, the associated risk factors in emergency gastrointestinal surgery remain unclear because emergency gastrointestinal surgery is rare and most of the previous studies have included small numbers of patients.^3^, ^4^, ^5^, ^6^, ^7^, ^8^, ^9^, ^10^, ^11^, ^12^, ^13^

The aims of this study were to investigate the impact of emergency surgery for gastrointestinal cancer on mortality and morbidity using the largest patient registry in Japan, to identify the risk factors for 30‐day mortality and incidence of overall postoperative complications in these patients who undergo emergency surgery.^15^

## 2 METHODS

### 2.1 Study design and setting

This retrospective observational study used data from the National Clinical Database (NCD), which is a nationwide surgical registration system in Japan that contains data on early clinical outcomes, including postoperative mortality and intraoperative and postoperative complications. The NCD is linked to the board certification system for gastrointestinal surgery and covers more than 95% of surgical cases in Japan.^15^, ^16^ Detailed data are collected for five types of major gastrointestinal surgery, and risk models for mortality and postoperative complications have been created in an effort to achieve better postoperative outcomes.^17^, ^18^, ^19^, ^20^, ^21^ We extracted the data for patients who underwent esophageal resection, total gastrectomy, distal gastrectomy, right hemicolectomy, or low anterior resection from 2012 to 2017 from this database. Data for patients aged ≥18 years who had a malignant tumor at the site of resection were included, and data for those with missing values for potential risk factors or outcome variables and those with an abnormal value for length of hospital stay (negative or zero) or body mass index (BMI) (<10 or ≥200) were excluded. Length of stay was defined as the time from the date of admission to the date of discharge. The study was approved by the Ethics Committee of Kyoto University.

### 2.2 Actual risk of emergency surgery for gastrointestinal cancer

We investigated the actual risk associated with each type of surgery by comparing the emergency surgery and non‐emergency surgery. Primary outcomes were 30‐day mortality and incidence of overall postoperative complications. We also set intraoperative outcomes, postoperative outcomes, and the incidence of each postoperative complication as secondary outcomes (Appendix S1).

### 2.3 Risk factors for 30‐day mortality and incidence of overall postoperative complications after emergency surgery

We focused on the patients who underwent emergency surgery and investigated the risk factors for the primary outcomes of the first analysis, namely, 30‐day mortality and incidence of overall postoperative complications for each type of emergency surgery. We analyzed both preoperative and intraoperative risk factors (Appendix S2).

### 2.4 Statistical analysis

Continuous variables are shown as the median and interquartile range and categorical variables are shown as the number and percentile. The risk of emergency surgery compared with non‐emergency surgery was calculated using a univariable logistic regression model and is reported as a crude odds ratio (OR) with a 95% confidence interval (CI) to demonstrate the actual risk of emergency surgery. The risk factors for 30‐day mortality and overall postoperative complications after emergency surgery were investigated first using univariable logistic regression model and then using a multivariable logistic regression model to identify independent risk factors. All factors included in the univariable analysis were considered to be clinically important and included as covariates in the multivariable analysis to identify true risk factors in the emergency surgery group. All *P*‐values were two‐sided and *P*‐values less than 0.05 were considered statistically significant. The statistical analyses were performed using R software (version 3.5.0, 2018; R Foundation for Statistical Computing, Vienna, Austria).

## 3 RESULTS

### 3.1 Esophageal resection

#### 3.1.1 Patient characteristics

**Figure 1:**
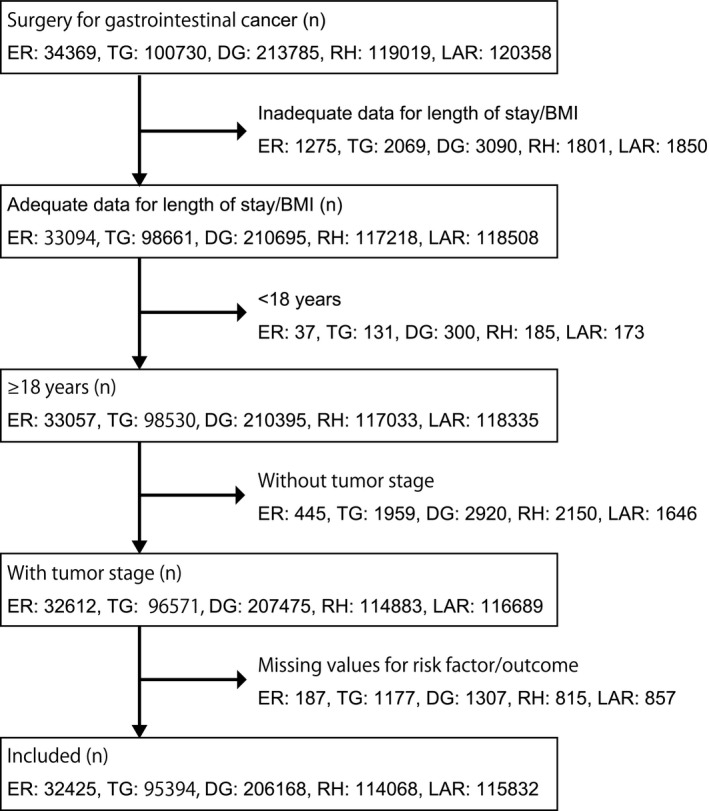
Flow diagram for patient selection. BMI, body mass index; DG, distal gastrectomy; ER, esophageal resection; LAR, low anterior resection; RH, right hemicolectomy; TG, total gastrectomy

A total of 32 425 patients who underwent esophageal resection for esophageal cancer (non‐emergency surgery, n = 32 315, 99.7%; emergency surgery, n = 110, 0.3%) were included in the study (Figure 1). The preoperative characteristics in the emergency surgery group included high proportions of patients with dyspnea, dependence in activities of daily living (ADL), metastatic cancer in another organ, weight loss, blood clotting defects, and sepsis. Their intraoperative characteristics included less use of thoracoscopy, high American Society of Anesthesiologists physical status (ASA‐PS), and advanced T stage (Table 1).

**Table 1:** Patient characteristics

Factor	Category	Esophageal resection (n = 32,425)		Total gastrectomy (n = 95,934)		Distal gastrectomy (n = 206,168)		Right hemicolectomy (n = 114,068)		Low anterior resection (n = 115,832)	
		Non‐emergency group (n = 32 315)	Emergency group (n = 110)	Non‐emergency group (n = 94 959)	Emergency group (n = 975)	Non‐emergency group (n = 204 609)	Emergency group (n = 1559)	Non‐emergency group (n = 109 169)	Emergency group (n = 4899)	Non‐emergency group (n = 114 784)	Emergency group (n = 1048)
		n (%)	n (%)	n (%)	n (%)	n (%)	n (%)	n (%)	n (%)	n (%)	n (%)
Preoperative factor											
Age (years)	70≤	13,135 (40.6)	42 (38.2)	53,416 (56.3)	558 (57.2)	114,284 (55.9)	1000 (64.1)	73,080 (66.9)	3297 (67.3)	49,617 (43.2)	498 (47.5)
Sex	Female	5408 (16.7)	14 (12.7)	24,409 (25.7)	214 (21.9)	67,869 (33.2)	484 (31.0)	55,118 (50.5)	2505 (51.1)	39,543 (34.4)	423 (40.4)
Body mass index	18.5≤, <25.0	22,137 (68.5)	74 (67.3)	63,998 (67.4)	626 (64.2)	13,8133 (67.5)	982 (63.0)	71,413 (65.4)	3044 (62.1)	76,684 (66.8)	690 (65.8)
	<18.5	6352 (19.7)	22 (20.0)	14,449 (15.2)	221 (22.7)	25,954 (12.7)	350 (22.5)	16,007 (14.7)	1203 (24.6)	13,231 (11.5)	203 (19.4)
	25.0≤	3826 (11.8)	14 (12.7)	16,512 (17.4)	128 (13.1)	40,522 (19.8)	227 (14.6)	21,749 (19.9)	652 (13.3)	24,869 (21.7)	155 (14.8)
Diabetes mellitus	+	4454 (13.8)	17 (15.5)	17,258 (18.2)	161 (16.5)	36,707 (17.9)	259 (16.6)	20,979 (19.2)	728 (14.9)	20,659 (18.0)	150 (14.3)
Smoking	+	24,069 (74.5)	78 (70.9)	44,880 (47.3)	442 (45.3)	90,092 (44.0)	628 (40.3)	30,491 (27.9)	1344 (27.4)	49,065 (42.7)	396 (37.8)
Habitual drinking	+	20,877 (64.6)	73 (66.4)	28,973 (30.5)	273 (28.0)	59,621 (29.1)	394 (25.3)	21,408 (19.6)	888 (18.1)	34,109 (29.7)	280 (26.7)
Dyspnea	+	405 (1.3)	4 (3.6)	1726 (1.8)	47 (4.8)	3551 (1.7)	62 (4.0)	2099 (1.9)	170 (3.5)	1319 (1.1)	24 (2.3)
Dependence in ADL	+	508 (1.6)	6 (5.5)	3825 (4.0)	147 (15.1)	9042 (4.4)	265 (17.0)	8655 (7.9)	1112 (22.7)	4057 (3.5)	115 (11.0)
Mechanical ventilation	+	43 (0.1)	3 (2.7)	37 (0.0)	20 (2.1)	78 (0.0)	17 (1.1)	54 (0.0)	30 (0.6)	43 (0.0)	3 (0.3)
COPD	+	2426 (7.5)	3 (2.7)	4728 (5.0)	41 (4.2)	9391 (4.6)	58 (3.7)	3394 (3.1)	142 (2.9)	3878 (3.4)	26 (2.5)
Pneumonia	+	217 (0.7)	1 (0.9)	304 (0.3)	11 (1.1)	733 (0.4)	29 (1.9)	457 (0.4)	81 (1.7)	279 (0.2)	9 (0.9)
Ascites	+	184 (0.6)	1 (0.9)	1861 (2.0)	94 (9.6)	2497 (1.2)	161 (10.3)	2877 (2.6)	683 (13.9)	1159 (1.0)	72 (6.9)
Esophageal varices	+	73 (0.2)	0 (0.0)	263 (0.3)	4 (0.4)	614 (0.3)	9 (0.6)	276 (0.3)	15 (0.3)	187 (0.2)	3 (0.3)
Hypertension	+	11,527 (35.7)	34 (30.9)	36,708 (38.7)	353 (36.2)	80,932 (39.6)	582 (37.3)	45,129 (41.3)	1722 (35.2)	40,467 (35.3)	317 (30.2)
Congestive heart failure	+	85 (0.3)	0 (0.0)	583 (0.6)	6 (0.6)	1454 (0.7)	21 (1.3)	1180 (1.1)	71 (1.4)	597 (0.5)	10 (1.0)
Angina pectoris	+	252 (0.8)	0 (0.0)	1377 (1.5)	11 (1.1)	2790 (1.4)	32 (2.1)	1442 (1.3)	59 (1.2)	1169 (1.0)	9 (0.9)
Symptomatic PVD	+	94 (0.3)	1 (0.9)	331 (0.3)	5 (0.5)	656 (0.3)	7 (0.4)	318 (0.3)	10 (0.2)	279 (0.2)	3 (0.3)
Acute renal failure	+	15 (0.0)	0 (0.0)	54 (0.1)	4 (0.4)	98 (0.0)	8 (0.5)	71 (0.1)	54 (1.1)	44 (0.0)	6 (0.6)
Dialysis	+	81 (0.3)	0 (0.0)	551 (0.6)	9 (0.9)	1590 (0.8)	26 (1.7)	762 (0.7)	45 (0.9)	561 (0.5)	4 (0.4)
History of CVD	+	819 (2.5)	2 (1.8)	3616 (3.8)	49 (5.0)	7640 (3.7)	81 (5.2)	4410 (4.0)	210 (4.3)	3504 (3.1)	34 (3.2)
Metastatic cancer	+	228 (0.7)	3 (2.7)	1974 (2.1)	101 (10.4)	2287 (1.1)	98 (6.3)	4333 (4.0)	338 (6.9)	3293 (2.9)	64 (6.1)
Long‐term steroid use	+	280 (0.9)	0 (0.0)	804 (0.8)	7 (0.7)	2014 (1.0)	17 (1.1)	1080 (1.0)	52 (1.1)	881 (0.8)	6 (0.6)
Weight loss	+	2430 (7.5)	13 (11.8)	6891 (7.3)	121 (12.4)	8927 (4.4)	160 (10.3)	4666 (4.3)	373 (7.6)	3184 (2.8)	59 (5.6)
Blood clotting defects	+	1010 (3.1)	9 (8.2)	3832 (4.0)	63 (6.5)	8319 (4.1)	115 (7.4)	4476 (4.1)	290 (5.9)	3528 (3.1)	42 (4.0)
Chemotherapy	+	6145 (19.0)	15 (13.6)	3352 (3.5)	72 (7.4)	2491 (1.2)	61 (3.9)	743 (0.7)	53 (1.1)	2548 (2.2)	11 (1.0)
Sepsis	+	47 (0.1)	2 (1.8)	93 (0.1)	48 (4.9)	154 (0.1)	109 (7.0)	244 (0.2)	482 (9.8)	153 (0.1)	88 (8.4)
Blood transfusion	+	234 (0.7)	2 (1.8)	2861 (3.0)	262 (26.9)	4969 (2.4)	273 (17.5)	3877 (3.6)	207 (4.2)	1175 (1.0)	34 (3.2)
Intraoperative factor											
Endoscopy	+	18,028 (55.8)	51 (46.4)	20,542 (21.6)	53 (5.4)	96,140 (47.0)	243 (15.6)	58,414 (53.5)	527 (10.8)	67,819 (59.1)	260 (24.8)
Diverting stoma	+	‐	‐	‐	‐	‐	‐	‐	‐	19,762 (17.2)	332 (31.7)
Concurrent surgery	+	9761 (30.2)	31 (28.2)	36,293 (38.2)	386 (39.6)	43,563 (21.3)	449 (28.8)	17,474 (16.0)	1228 (25.1)	30,500 (26.6)	464 (44.3)
ASA‐PS	1,2	29,806 (92.2)	90 (81.8)	84,415 (88.9)	584 (59.9)	181,930 (88.9)	1000 (64.1)	94,128 (86.2)	3283 (67.0)	104,356 (90.9)	775 (74.0)
	3,4	2479 (7.7)	19 (17.3)	10,477 (11.0)	367 (37.6)	22,574 (11.0)	532 (34.1)	14,985 (13.7)	1545 (31.5)	10 374 (9.0)	263 (25.1)
	5	30 (0.1)	1 (0.9)	67 (0.1)	24 (2.5)	105 (0.1)	27 (1.7)	56 (0.1)	71 (1.4)	54 (0.0)	10 (1.0)
T	T3≤	15,037 (46.5)	64 (58.2)	56,445 (59.4)	793 (81.3)	68,776 (33.6)	1000 (64.1)	82,770 (75.8)	4550 (92.9)	72,645 (63.3)	883 (84.3)
N	N1≤	17,464 (54.0)	65 (59.1)	50,898 (53.6)	690 (70.8)	71,287 (34.8)	895 (57.4)	48 938 (44.8)	2741 (56.0)	47,856 (41.7)	552 (52.7)
M	M1	875 (2.7)	4 (3.6)	10,114 (10.7)	265 (27.2)	10,040 (4.9)	297 (19.1)	12,932 (11.8)	912 (18.6)	10,683 (9.3)	176 (16.8)
Residual tumor	R1≤	2013 (6.2)	8 (7.3)	10,384 (10.9)	303 (31.1)	10,926 (5.3)	336 (21.6)	8812 (8.1)	828 (16.9)	6714 (5.8)	139 (13.3)

Abbreviations: ADL, activities of daily living; ASA‐PS, American Society of Anesthesiologists physical status; COPD, chronic obstructive pulmonary disease; CVD, cerebrovascular disease; PVD, peripheral vascular disease.

#### 3.1.2 Actual risk of emergency surgery

There were no deaths and only a small number of postoperative complications in the emergency surgery group (Table S1). Therefore, we did not calculate the ORs for emergency esophageal resection in comparison with non‐emergency surgery.

### 3.2 Total gastrectomy

#### 3.2.1 Patient characteristics

A total of 95 934 patients underwent total gastrectomy for gastric cancer (Figure 1). Non‐emergency surgery was performed in 94 959 patients (99.0%) and emergency surgery was performed for 975 patients (1.0%). The preoperative characteristics in the emergency surgery group included high proportions of patients with low BMI, dyspnea, dependence in ADL, ascites, metastatic cancer in another organ, weight loss, blood clotting defects, and sepsis. Preoperative blood transfusion and chemotherapy were more common in the emergency surgery group. Intraoperative characteristics included less use of laparoscopy, high ASA‐PS, advanced TNM stages, and a high frequency of residual tumor (Table 1).

#### 3.2.2 Actual risk of emergency surgery

The primary outcome of 30‐day mortality was significantly higher in the emergency surgery group than in the non‐emergency surgery group (OR: 6.12, 95% CI: 4.50‐8.32, *P* < .001). The incidence of overall postoperative complications was also significantly higher in the emergency surgery group (OR: 1.68, 95% CI: 1.48‐1.91, *P* < .001) (Table 2).

**Table 2:** Primary outcomes

Factor	Total gastrectomy			Distal gastrectomy			Right hemicolectomy			Low anterior resection		
	OR	95% CI	*P*‐value	OR	95% CI	*P*‐value	OR	95% CI	*P*‐value	OR	95% CI	*P*‐value
30‐day mortality	±6.12	4.50‐8.32	<.001	8.63	6.65‐11.18	<.001	6.98	5.87‐8.30	<.001	4.86	2.98‐7.93	<.001
Overall postoperative complications	±1.68	1.48‐1.91	<.001	1.78	1.61‐1.98	<.001	2.18	2.05‐2.31	<.001	1.70	1.50‐1.93	<.001

Abbreviations: CI, confidence interval; OR, odds ratio.

For the secondary outcomes, incidence of intraoperative and postoperative adverse events was significantly higher in the emergency surgery group (Table 3). The frequencies of intraoperative and postoperative blood transfusion and mechanical ventilation and the incidence of pulmonary embolism, renal dysfunction, prolonged disturbance of consciousness, and deep vein thrombosis were particularly high in the emergency surgery group (OR > 3; Table 3). The number of patients with each complication is listed in Table S1.

**Table 3:** Secondary outcomes (categorical variables)

Factor	Total gastrectomy			Distal gastrectomy			Right hemicolectomy			Low anterior resection		
	OR	95% CI	*P*‐value	OR	95% CI	*P*‐value	OR	95% CI	*P*‐value	OR	95% CI	*P*‐value
Intraoperative outcomes												
Blood transfusion	±6.78	5.97‐7.70	<.001	7.31	6.58‐8.12	<.001	3.57	3.33‐3.82	<.001	3.55	3.04‐4.14	<.001
Adverse events	±2.51	1.47‐4.27	.001	1.21	0.60‐2.43	.591	1.84	1.30‐2.62	<.001	1.60	0.83‐3.10	.232
Postoperative outcomes												
Unscheduled intratracheal intubation	±2.62	1.82‐3.77	<.001	3.13	2.21‐4.44	<.001	3.95	3.15‐4.97	<.001	2.57	1.51‐4.38	<.001
Mechanical ventilation	±4.31	3.22‐5.78	<.001	5.97	4.57‐7.81	<.001	10.43	8.94‐12.16	<.001	7.43	5.36‐10.29	<.001
Blood transfusion	±4.70	3.89‐5.69	<.001	4.54	3.75‐5.50	<.001	3.91	3.48‐4.39	<.001	3.93	3.03‐5.09	<.001
ICU admission	±1.84	1.62‐2.09	<.001	1.72	1.56‐1.91	<.001	1.86	1.75‐1.97	<.001	1.43	1.26‐1.61	<.001
Re‐operation	±1.72	1.30‐2.28	<.001	2.27	1.78‐2.89	<.001	1.85	1.62‐2.12	<.001	1.19	0.95‐1.49	.146
Re‐admission	±1.06	0.73‐1.53	.759	1.47	1.12‐1.93	.006	1.18	1.01‐1.38	.037	1.42	1.05‐1.94	.030
Postoperative complications												
Superficial incisional SSI	±2.32	1.75‐3.07	<.001	3.34	2.69‐4.15	<.001	2.28	2.06‐2.52	<.001	2.77	2.22‐3.46	<.001
Deep incisional SSI	±1.72	1.06‐2.79	.028	2.46	1.64‐3.69	<.001	3.36	2.85‐3.96	<.001	2.91	2.05‐4.13	<.001
Deep SSI	±1.11	0.84‐1.47	.450	1.34	1.02‐1.77	.035	2.31	1.98‐2.70	<.001	1.32	1.05‐1.66	.017
Wound disruption	±2.46	1.41‐4.28	.001	3.43	2.22‐5.31	<.001	3.66	2.99‐4.49	<.001	2.94	1.78‐4.85	<.001
Anastomotic leakage	±1.20	0.90‐1.59	.212	1.55	1.18‐2.05	.002	1.69	1.41‐2.03	<.001	0.96	0.77‐1.19	.701
Pancreatic fistula	±0.78	0.56‐1.08	.139	1.14	0.84‐1.56	.393	0.74	0.35‐1.57	.431	2.67	0.66‐10.89	.170
Pneumonia	±1.45	1.08‐1.95	.013	2.71	2.17‐3.39	<.001	3.96	3.42‐4.59	<.001	2.66	1.73‐4.08	<.001
Pulmonary embolism	±3.19	1.31‐7.80	.011	3.04	1.25‐7.40	.014	1.79	0.91‐3.54	.092	0.00	0.00‐1.4 × 10^277^	.969
Renal dysfunction	±3.83	2.69‐5.46	<.001	4.42	3.25‐6.01	<.001	5.36	4.49‐6.39	<.001	3.16	2.16‐4.62	<.001
Urinary infection	±1.65	0.85‐3.19	.139	2.63	1.66‐4.15	<.001	2.50	1.93‐3.23	<.001	0.72	0.37‐1.40	.335
CNS dysfunction	±2.41	1.07‐5.44	.034	3.19	1.70‐5.99	<.001	2.76	1.85‐4.12	<.001	2.97	1.22‐7.23	.017
Prolonged disturbance of consciousness	±4.12	2.31‐7.37	<.001	5.15	3.07‐8.65	<.001	8.66	6.61‐11.34	<.001	4.35	2.04‐9.29	<.001
Cardiac arrest	±2.89	1.58‐5.29	<.001	4.01	2.40‐6.73	<.001	4.16	2.95‐5.87	<.001	4.49	2.30‐8.77	<.001
Acute myocardial infarction	±2.27	0.56‐9.23	.253	2.82	0.90‐8.85	.076	2.95	1.47‐5.92	.002	5.78	1.81‐18.48	.003
Deep vein thrombosis	±3.96	2.10‐7.47	<.001	3.15	1.56‐6.35	.001	1.90	1.23‐2.93	.004	2.17	0.89‐5.27	.087
Sepsis	±2.86	2.18‐3.75	<.001	4.26	3.35‐5.42	<.001	7.37	6.48‐8.38	<.001	3.51	2.73‐4.51	<.001

Abbreviations: CI, confidence interval; CNS, central nervous system; ICU, intensive care unit; NS, not significant; OR, odds ratio; SSI, surgical site infection.

Compared with the non‐emergency surgery group, the emergency surgery group had significantly shorter anesthesia and operating times (335 minutes vs 295 minutes, *P* < .001 and 277 vs 237 minutes, *P* < .001, respectively), significantly higher estimated blood loss (290 mL vs 484 mL, *P* < .001), and significantly longer hospital stay (21 days vs 25 days, *P* < .001; Table 4).

**Table 4:** Secondary outcomes (continuous variables)

Factor		Total gastrectomy					Distal gastrectomy					Right hemicolectomy					Low anterior resection				
		Non‐emergency group (n = 94 959)		Emergency group (n = 975)		*P*‐value	Non‐emergency group (n = 204 609)		Emergency group (n = 1559)		*P*‐value	Non‐emergency group (n = 109 169)		Emergency group (n = 4899)		*P*‐value	Non‐emergency group (n = 114 784)		Emergency group (n = 1048)		*P*‐value
Intraoperative outcomes																					
Anesthesia time (min)	Median [IQR]	335	[271, 411]	295	[240, 360]	<.001	312	[253, 380]	265	[210, 330]	<.001	256	[205, 319]	220	[180, 266]	<.001	330	[262, 417]	275	[220, 353]	<.001
Operating time (min)	Median [IQR]	277	[219, 348]	237	[188, 300]	<.001	255	[200, 317]	208	[160, 272]	<.001	198	[152, 255]	165	[130, 209]	<.001	264	[202, 344]	212	[165, 284]	<.001
Estimated blood loss (mL)	Median [IQR]	290	[120, 565]	484	[215, 924]	<.001	110	[30, 280]	215	[90, 500]	<.001	61	[20, 180]	160	[50, 380]	<.001	75	[15, 250]	225	[52, 510]	<.001
Postoperative outcomes																					
Length of hospital stay (days)	Median [IQR]	21	[16, 32]	25	[17, 40]	<.001	18	[14, 26]	22	[16, 36]	<.001	18	[14, 27]	21	[15, 33]	<.001	20	[15, 31]	25	[18, 40]	<.001

Abbreviation: IQR: interquartile range.

### 3.3 Distal gastrectomy

#### 3.3.1 Patient characteristics

A total of 206 168 patients underwent distal gastrectomy for gastric cancer (non‐emergency surgery, n = 204 609, 99.2%; emergency surgery, n = 1559, 0.8%; Figure 1). The preoperative characteristics in the emergency surgery group included high proportions of elderly patients and patients with low BMI, dyspnea, dependence in ADL, ascites, metastatic cancer in another organ, weight loss, blood clotting defects, and sepsis. Preoperative blood transfusion and chemotherapy were more frequent in the emergency surgery group. The intraoperative characteristics in the emergency surgery group included less use of laparoscopy, a high frequency of concurrent surgery, high ASA‐PS, advanced TNM stage, and a high frequency of residual tumor (Table 1).

#### 3.3.2 Actual risk of emergency surgery

The 30‐day mortality was significantly higher in the emergency surgery group than in the non‐emergency surgery group (OR: 8.63, 95% CI: 6.65‐11.18, *P* < .001). The incidence of overall postoperative complications was also significantly higher in the emergency surgery group (OR: 1.78, 95% CI: 1.61‐1.98, *P* < .001) (Table 2).

For the secondary outcomes, the incidence of intraoperative and postoperative adverse events was higher in the emergency surgery group (Table 3). The frequencies of intraoperative and postoperative blood transfusion, unscheduled intratracheal intubation, and mechanical ventilation and the incidence of superficial incisional surgical site infection (SSI), wound disruption, pulmonary embolism, renal dysfunction, central nervous system (CNS) dysfunction, prolonged disturbance of consciousness, cardiac arrest, deep vein thrombosis, and sepsis was higher in the emergency surgery group (OR > 3; Table 3). The numbers of patients with each complication are shown in Table S1.

Compared with the non‐emergency surgery group, the emergency surgery group had significantly shorter anesthesia and operating times (312 vs 265 minutes, *P* < .001 and 255 vs 208 minutes, *P* < .001, respectively), significantly greater estimated blood loss (110 vs 215 mL, *P* < .001), and a significantly longer hospital stay (18 vs 22 days, *P* < .001; Table 4).

### 3.4 Right hemicolectomy

#### 3.4.1 Patient characteristics

The data for the 114 068 patients in the database who underwent right hemicolectomy for colon cancer were included in the analysis; 109 169 (95.7%) of these patients underwent non‐emergency surgery and 4899 (4.3%) underwent emergency surgery (Figure 1). Analysis of the preoperative characteristics in the emergency surgery group revealed a high frequency of low BMI, dyspnea, dependence in ADL, ascites, metastatic cancer in another organ, weight loss, blood clotting defects, and sepsis. Intraoperative characteristics included less use of laparoscopy, a high frequency of concurrent surgery, poor ASA‐PS, advanced TNM stages, and a high frequency of residual tumor (Table 1).

#### 3.4.2 Actual risk of emergency surgery

The 30‐day mortality was significantly higher in the emergency surgery group than in the non‐emergency surgery group (OR: 6.98, 95% CI: 5.87‐8.30, *P* < .001). The incidence of overall postoperative complications was also significantly higher in the emergency surgery group (OR: 2.18, 95% CI: 2.05‐2.31, *P* < .001) (Table 2).

The incidence of many intraoperative and postoperative adverse events was higher in the emergency surgery group than in the non‐emergency surgery group (Table 3). The frequencies of intraoperative and postoperative blood transfusion, unscheduled intratracheal intubation, and mechanical ventilation and the incidence of deep incisional SSI, wound disruption, pneumonia, renal dysfunction, prolonged disturbance of consciousness, cardiac arrest, and sepsis was particularly high in the emergency surgery group (OR > 3; Table 3). The numbers of patients with each complication are shown in Table S1.

Compared with the non‐emergency surgery group, the emergency surgery group had significantly shorter anesthesia time and operating times (256 minutes vs 220 minutes, *P* < .001 and 198 minutes vs 165 minutes, *P* < .001, respectively), significantly higher estimated blood loss (61 mL vs 160 mL, *P* < .001), and a significantly longer hospital stay (18 days vs 21 days, *P* < .001; Table 4).

### 3.5 Low anterior resection

#### 3.5.1 Patient characteristics

A total of 115 832 patients underwent low anterior resection for rectal cancer (non‐emergency surgery, n = 114 784, 99.1%; emergency surgery group, n = 1048, 0.9%; Figure 1). The preoperative characteristics in the emergency surgery group included high proportions of low BMI, dyspnea, dependence in ADL, ascites, metastatic cancer in another organ, weight loss, and sepsis. Preoperative blood transfusion was more frequent in the emergency surgery group. The intraoperative characteristics in this group included less use of laparoscopy, more cases of diverting stoma, concurrent surgery, higher ASA‐PS, a more advanced TNM stage, and a higher frequency of residual tumor (Table 1).

#### 3.5.2 Actual risk of emergency surgery

The 30‐day mortality was significantly higher in the emergency surgery group than in the non‐emergency surgery group (OR: 4.86, 95% CI: 2.98‐7.93, *P* < .001). The overall incidence of postoperative complications was also significantly higher in the emergency surgery group (OR: 1.70, 95% CI: 1.51‐1.93, *P* < .001) (Table 2).

Intraoperative and postoperative adverse events were more frequent in the emergency surgery group than in the non‐emergency surgery group (Table 3). The frequencies of intraoperative and postoperative blood transfusion and mechanical ventilation and the incidence of renal dysfunction, prolonged disturbance of consciousness, cardiac arrest, acute myocardial infarction, and sepsis was particularly high in the emergency surgery group (OR > 3; Table 3). The numbers of patients with each complication are listed in Table S1.

Compared with the non‐emergency surgery group, the emergency surgery group had significantly shorter anesthesia and operating times (330 minutes vs 275 minutes, *P* < .001 and 264 minutes vs 212 minutes, *P* < .001, respectively), a significantly higher estimated blood loss (75 mL vs 225 mL, *P* < .001), and a significantly longer hospital stay (20 days vs 25 days, *P* < .001; Table 4).

### 3.6 Risk factors in the emergency surgery group

#### 3.6.1 Risk factors for 30‐day mortality

We did not analyze the risk factors for 30‐day mortality after esophageal resection because no patients in this group died. Moreover, the numbers of events were too small in the groups that underwent the other four types of emergency surgery to perform multivariable analyses; therefore, only univariable analysis was performed (Table S2). Several factors were identified as potentially critical (OR > 3) for 30‐day mortality (Appendix 3).

#### 3.6.2 Risk factors for postoperative overall complications

The risk factors for postoperative overall complications were not analyzed in the esophageal resection group on account of the numbers of complications being too small. The results of the univariable analysis of risk factors for the other four types of surgery are shown in Table S3. Multivariable analyses identified the following independent factors as critical (OR > 3) for each type of surgery: low BMI, history of cerebrovascular disease (CVD), preoperative sepsis, and high ASA‐PS in the total gastrectomy group; being elderly, habitual drinking, history of CVD, concurrent surgery, and high ASA‐PS in the distal gastrectomy group; being elderly, male sex, dependence in ADL, chronic obstructive pulmonary disease (COPD), preoperative sepsis, concurrent surgery, and high ASA‐PS in the right hemicolectomy group; and high BMI, weight loss, preoperative sepsis, high ASA‐PS, and advanced T stage in the low anterior resection group (Table 5).

**Table 5:** Risk factors for overall postoperative complications: multivariable analysis

Factor	Category	Total gastrectomy			Distal gastrectomy			Right hemicolectomy			Low anterior resection		
		OR	95% CI	*P*‐value	OR	95% CI	*P*‐value	OR	95% CI	*P*‐value	OR	95% CI	*P*‐value
Preoperative factor													
Age (years)	70≤/<70	1.11	0.83‐1.49	.487	1.31	1.01‐1.69	.041	1.17	1.01‐1.35	.037	0.97	0.73‐1.30	.847
Sex	Female/Male	0.91	0.63‐1.30	.593	0.76	0.58‐1.01	.059	0.75	0.65‐0.86	<.001	0.76	0.56‐1.03	.075
Body mass index	18.5≤, <25.0	Reference			Reference			Reference			Reference		
	<18.5	1.60	1.15‐2.23	.006	1.12	0.85‐1.50	.419	1.11	0.96‐1.29	.159	1.18	0.83‐1.67	.365
	25.0≤	1.13	0.75‐1.71	.567	1.36	0.98‐1.88	.067	1.17	0.97‐1.41	.094	1.75	1.19‐2.56	.004
Diabetes mellitus	±	0.89	0.62‐1.30	.553	1.08	0.79‐1.47	.620	1.01	0.85‐1.21	.899	1.19	0.81‐1.74	.375
Smoking	±	1.04	0.77‐1.41	.802	1.19	0.92‐1.55	.193	1.16	0.99‐1.36	.070	1.22	0.90‐1.67	.204
Habitual drinking	±	0.95	0.69‐1.31	.757	0.70	0.52‐0.94	.018	0.97	0.81‐1.16	.758	1.25	0.90‐1.74	.184
Dyspnea	±	1.09	0.56‐2.13	.804	1.41	0.79‐2.54	.246	1.13	0.78‐1.63	.511	0.46	0.16‐1.34	.154
Dependence in ADL	±	1.11	0.74‐1.67	.621	1.16	0.85‐1.59	.339	1.46	1.25‐1.70	<.001	1.51	0.95‐2.39	.078
Mechanical ventilation	±	1.38	0.52‐3.66	.522	0.77	0.25‐2.35	.645	0.96	0.42‐2.16	.918	3.91	0.29‐52.52	.304
COPD	±	1.37	0.69‐2.73	.367	1.13	0.62‐2.04	.696	1.51	1.03‐2.21	.034	2.18	0.83‐5.77	.115
Pneumonia	±	1.29	0.30‐5.57	.730	1.59	0.71‐3.56	.263	1.02	0.62‐1.65	.949	3.92	0.56‐27.57	.170
Ascites	±	1.07	0.66‐1.73	.783	1.25	0.85‐1.82	.253	1.18	0.98‐1.41	.076	1.09	0.64‐1.86	.761
Esophageal varices	±	0.55	0.06‐5.44	.609	1.05	0.24‐4.62	.950	3.02	0.98‐9.27	.054	0.79	0.05‐12.93	.872
Hypertension	±	1.12	0.83‐1.52	.442	1.06	0.83‐1.35	.640	1.16	1.01‐1.32	.036	0.99	0.73‐1.34	.937
Congestive heart failure	±	1.40	0.12‐16.27	.787	1.76	0.66‐4.68	.257	1.41	0.83‐2.38	.207	2.29	0.44‐12.04	.328
Angina pectoris	±	3.71	0.82‐16.69	.088	1.52	0.70‐3.29	.292	1.40	0.79‐2.51	.253	2.68	0.42‐17.16	.298
Symptomatic PVD	±	0.51	0.07‐3.99	.521	0.40	0.06‐2.65	.345	5.05	0.58‐43.90	.142	1.32	0.05‐32.26	.865
Acute renal failure	±	0.85	0.06‐11.49	.901	3.82	0.72‐20.39	.116	1.18	0.64‐2.20	.592	1.06	0.10‐10.90	.960
Dialysis	±	1.63	0.35‐7.54	.531	1.48	0.63‐3.50	.373	1.25	0.66‐2.40	.494	2.66	0.23‐31.31	.437
History of CVD	±	2.59	1.34‐5.01	.005	1.64	1.00‐2.68	.050	1.12	0.83‐1.53	.453	0.99	0.44‐2.21	.973
Metastatic cancer	±	1.08	0.65‐1.80	.760	1.25	0.76‐2.06	.380	1.05	0.80‐1.38	.721	0.73	0.37‐1.41	.345
Long‐term steroid use	±	3.61	0.64‐20.54	.147	2.67	0.95‐7.54	.064	1.12	0.62‐2.00	.715	2.41	0.37‐15.59	.356
Weight loss	±	1.13	0.73‐1.74	.583	0.81	0.54‐1.20	.292	1.22	0.97‐1.55	.089	3.17	1.67‐6.03	<.001
Blood clotting defects	±	1.49	0.81‐2.74	.196	1.25	0.81‐1.94	.316	0.88	0.67‐1.16	.356	2.17	0.99‐4.78	.053
Chemotherapy	±	0.79	0.45‐1.36	.391	0.93	0.51‐1.67	.797	1.05	0.58‐1.89	.875	0.68	0.18‐2.54	.563
Sepsis	±	1.98	1.02‐3.83	.043	1.31	0.82‐2.08	.258	1.78	1.43‐2.21	<.001	2.06	1.22‐3.47	.007
Blood transfusion	±	1.25	0.90‐1.74	.187	1.35	0.99‐1.83	.058	1.09	0.80‐1.48	.592	0.93	0.42‐2.04	.851
Intraoperative factor													
Endoscopy	±	1.04	0.55‐1.96	.901	0.80	0.56‐1.15	.222	0.58	0.46‐0.72	<.001	1.09	0.78‐1.52	.615
Diverting stoma	±	‐			‐			‐			1.27	0.81 1.98	.299
Concurrent surgery	±	1.14	0.86‐1.51	.364	1.35	1.06‐1.72	.016	1.74	1.51‐2.01	<.001	1.00	0.66‐1.53	.988
ASA‐PS	1,2	Reference			Reference			Reference			Reference		
	3,4	1.21	0.90‐1.63	.208	1.26	0.97‐1.62	.079	1.57	1.37‐1.80	<.001	1.69	1.21‐2.35	.002
	5	3.44	1.34‐8.80	.010	5.70	2.14‐15.19	<.001	2.70	1.57‐4.64	<.001	5.62	1.07‐29.43	.041
T	T3≤/≤T2	1.41	0.90‐2.20	.131	1.22	0.88‐1.69	.223	1.29	0.99‐1.68	.059	1.62	1.06‐2.46	.026
N	N1≤/N0	1.06	0.73‐1.52	.774	1.16	0.87‐1.56	.314	0.99	0.87‐1.13	.894	1.00	0.75‐1.33	.990
M	M1/M0	0.87	0.58‐1.32	.522	1.04	0.71‐1.52	.842	0.94	0.76‐1.17	.602	0.85	0.54‐1.33	.481
Residual tumor	R1≤/R0	0.91	0.62‐1.34	.646	0.98	0.69‐1.41	.925	1.19	0.97‐1.46	.104	1.49	0.93‐2.39	.096

Abbreviations: ASA‐PS: American Society of Anesthesiologists physical status, ADL: activities of daily living, COPD: chronic obstructive pulmonary disease, CVD: cerebrovascular disease, PVD: peripheral vascular disease.

## 4 DISCUSSION

In this study, we investigated the frequencies of five types of major emergency surgery for gastrointestinal cancer (for which emergency surgeries accounted for 0.3%‐4.3% of all surgeries) and calculated the actual 30‐day mortality and incidence of complications associated with all of these operations except for esophageal resection. Our results show that the risks of these four types of gastrointestinal surgeries were significantly higher when they were performed on an emergency basis. However, these findings do not imply that emergency surgeries should be avoided. Emergency surgery is usually performed only in sudden‐onset cases and cases where surgery is unavoidable, such as peritonitis as a result of gastrointestinal perforation and intestinal stenosis. The risks of emergency surgery in this study included not only the risk of surgery per se but also the risk of the critical conditions requiring emergency treatment. Emergency surgery should not be avoided unnecessarily; however, knowledge of the actual risks of emergency surgery in critically ill patients is important for postoperative management.

We analyzed actual risks of mortality and postoperative complications in the former part of this study. Many studies reported that emergency surgery was a risk factor in gastrointestinal surgeries^3^, ^4^, ^5^, ^6^, ^7^, ^8^, ^9^, ^10^, ^11^, ^12^, ^13^, ^18^, ^19^, ^20^, ^21^. Our results corresponded to the previous studies and made the risk of emergency surgery robust. In the latter part of this study, we extracted patients undergoing emergency surgery and analyzed risk factors for postoperative complications. Emergency surgery is rare and the risk of postoperative complications after emergency surgery is not fully assessed. It is a novel point of this study to show the risk factors for postoperative complications in patients undergoing emergency surgeries using the nationwide database.

As in previous studies using NCD^17^, ^18^, ^19^, ^20^, ^21^, the risk calculator to detect high‐risk patients may be useful for surgeons. However, the main purpose of this study is to show information about the actual mortality and morbidity of emergency surgery and the risk factors for overall complications in patients undergoing emergency surgery. Emergency patients with any risk factor are at super high risk because emergency surgery itself is a risk factor. Emergency patients are often in critical and complicated conditions, so we think that surgeons need informative references rather than a simple risk score. Also, the individual correlations between preoperative morbidity and postoperative complication are interesting. However, study results would be numerous and difficult to understand if all individual correlations are shown. Patients undergoing emergency surgery are often in poor condition and any complication would be critical for them. Thus, we showed the risk factors for overall complications in emergency patients.

The incidence of unscheduled intratracheal intubation, mechanical ventilation, admission to the intensive care unit, superficial and deep incisional SSIs, wound disruption, pneumonia, renal dysfunction, CNS dysfunction, prolonged disturbance of consciousness, cardiac arrest, and sepsis was significantly higher when surgery was performed on an emergency basis. In contrast, incidence of pulmonary embolism and deep vein thrombosis was high in patients who underwent emergency total or distal gastrectomy, and incidence of acute myocardial infarction was high in those who underwent emergency colorectal surgery. The incidence of intraoperative adverse events was high in patients who underwent emergency total gastrectomy or right hemicolectomy, in those who required repeat operations other than low anterior resection, and in those who were re‐admitted for emergency surgery other than total gastrectomy. These findings suggest two types of complications, that is, complications that are common in emergency surgery, which are mainly attributable to the burden of the indication for emergency surgery, and complications specific to particular types of surgery, which are due to a combination of the burdens of emergency surgery and the indication for emergency surgery. Knowledge of the frequency of each complication might facilitate early detection and prevent any complications that occur from increasing severity.

The anesthesia and operating times were significantly shorter for all four types of emergency surgery; however, estimated blood loss was significantly greater and length of hospital stay was significantly longer in the emergency surgery group than in the non‐emergency surgery group. Surgeons generally try to complete emergency surgery as rapidly as possible because patients who need such surgery are often in a poor state with conditions such as sepsis and clotting abnormalities. Less use of laparoscopy in the emergency surgery groups might be influential. In general, a shorter operating time leads to a lower physical burden on the patient, a lower incidence of complications, and a shorter hospital stay^22^, ^23^. However, in this study, the emergency operating times were shorter but with a high incidence of complications and longer length of hospital stay, indicating that patients who need emergency surgery are in an intrinsically poorer condition regardless of the physical burden of the surgery. Although emergency surgery was performed in a shorter time to minimize the physical burden, the patients had higher 30‐day mortality (OR: 4.86‐8.63; all *P* < .001) and higher incidence of overall postoperative complications (OR: 1.68‐2.18; all *P* < .001).

Male sex and sepsis or high ASA‐PS tended to be associated with high risk of overall postoperative complications. Chemotherapy before emergency surgery and cancer progress, excluding low anterior resection for advanced T stage disease, did not influence the overall postoperative complications. Patients with low BMI in total gastrectomy and those with weight loss in low anterior resection were at high risk for the postoperative complications. Low BMI and weight loss often reflect malnutrition and are known to be risk factors for postoperative complications in various surgeries.^24^, ^25^, ^26^, ^27^ Patients undergoing total gastrectomy or low anterior resection might be more sensitive to the nutrition status than those undergoing another procedure in emergency cases. Dependence in ADL and COPD were associated with high risk in colorectal surgery, and a history of CVD was associated with high risk in gastrectomy. Patients who underwent concurrent surgery with distal gastrectomy or right hemicolectomy were also at high risk. Diverting stoma was not associated with the incidence of overall postoperative complications in low anterior resection. These results suggest that being in a poor state preoperatively was more critical for patients undergoing emergency surgery than cancer progression or the effects of preoperative chemotherapy. As in the above description about the incidence of postoperative complications, some risk factors were common across the four types of emergency surgery and others were different, suggesting that all emergency gastrointestinal surgeries involve a physical burden but that some have an additional burden depending on the type of surgery.

The main strength of this study is that it used nationwide data in Japan. The NCD database covers almost all surgeries performed in Japan because it is linked to the board certification system in Japan. Moreover, many of the variables studied, including preoperative factors and postoperative complications, have been collected for five types of surgery. Emergency surgery is rare and only a large‐scale database like the NCD can clarify its actual risks. Furthermore, only patients with cancer were included in this study, which eliminated the heterogeneity between benign and malignant diseases. We also included data for the five types of major surgery performed for gastrointestinal cancer to be able to provide gastrointestinal surgeons with useful information regarding the risks of these surgeries when performed on an emergency basis. However, the study also has some limitations, particularly its retrospective design and the potential for recall bias, and transcription errors. Also, data input was dependent on each institution. Nevertheless, we believe that the influence of theses biases was limited because the data were entered into the NCD on a yearly basis and it has been reported that there is little difference between the data on medical charts and those in the NCD.^28^

In conclusion, emergency surgery for gastrointestinal cancer was associated with high 30‐day mortality and morbidity in Japan. Emergency surgery had an additional burden on patients depending on the type of surgery. The actual risk of emergency surgery and the risk factors for overall postoperative complications in emergency cases are shown to serve as a reference for postoperative management.

## DISCLOSURE

Funding: This study was supported by a grant from the Japanese Society for Abdominal Emergency Medicine.

Conflict of Interest: Hideki Endo, Nao Ichihara, and Hiroaki Miyata are affiliated with the Department of Healthcare Quality Assessment, which is a social collaboration department at the University of Tokyo supported by National Clinical Database, Johnson & Johnson KK, and Nipro Corporation. Yuko Kitagawa has received research expenses or scholarship donations from Chugai Pharmaceutical Co., Ltd., Taiho Pharmaceutical Co., Ltd. For the remaining authors, none were declared. The funding for this study was provided by the Japanese Society for Abdominal Emergency Medicine. The funding source had no role in the design, practice, or analysis of this study.

Ethical Statements: The protocol for this research project has been approved by the Ethics Committee of Kyoto University (Approval No. R1779).

## Supporting information

Table S1

Table S2

Table S3

Supplementary Material

Supplementary Material

Supplementary Material
