# Supplementary material for: Emergency surgery for gastrointestinal cancer: A nationwide study in Japan based on the National Clinical Database
Source: Ann Gastroenterol Surg. 2020 Jun 21;4(5):549–61. doi: 10.1002/ags3.12353 (PMC7511565; doi:10.1002/ags3.12353)
Supplement: Supplementary file 1 — Table S1 [file AGS3-4-549-s001.docx]

| **Table S1** Incidence of each outcome | | | | | | | | | | | | | | | |
| --- | --- | --- | --- | --- | --- | --- | --- | --- | --- | --- | --- | --- | --- | --- | --- |
|  |  | Esophageal resection (n = 32,425) | |  | Total gastrectomy (n = 95,934) | |  | Distal gastrectomy (n = 206,168) | |  | Right hemicolectomy (n = 114,068) | |  | Low anterior resection (n = 115,832) | |
| Factor | Category | Non-emergency group | Emergency group |  | Non-emergency group | Emergency group |  | Non-emergency group | Emergency group |  | Non-emergency group | Emergency group |  | Non-emergency group | Emergency group |
|  |  | (n = 32,315) | (n = 110) |  | (n = 94,959) | (n = 975) |  | (n = 204,609) | (n = 1559) |  | (n = 109,169) | (n = 4899) |  | (n = 114,784) | (n = 1048) |
|  |  | n (%) | n (%) |  | n (%) | n (%) |  | n (%) | n (%) |  | n (%) | n (%) |  | n (%) | n (%) |
| Primary outcomes |  |  |  |  |  |  |  |  |  |  |  |  |  |  |  |
| 30-day mortality | + | 294 (0.9) | 0 (0.0) |  | 745 (0.8) | 45 (4.6) |  | 994 (0.5) | 63 (4.0) |  | 566 (0.5) | 172 (3.5) |  | 388 (0.3) | 17 (1.6) |
| Overall postoperative complications | + | 16,425 (50.8) | 56 (50.9) |  | 27,491 (29.0) | 396 (40.6) |  | 44,512 (21.8) | 517 (33.2) |  | 23,604 (21.6) | 1838 (37.5) |  | 32,176 (28.0) | 418 (39.9) |
|  |  |  |  |  |  |  |  |  |  |  |  |  |  |  |  |
| Intraoperative outcomes |  |  |  |  |  |  |  |  |  |  |  |  |  |  |  |
| Blood transfusion | + | 5766 (17.8) | 25 (22.7) |  | 14,393 (15.2) | 534 (54.8) |  | 14,762 (7.2) | 565 (36.2) |  | 8801 (8.1) | 1167 (23.8) |  | 7198 (6.3) | 201 (19.2) |
| Adverse events | + | 380 (1.2) | 2 (1.8) |  | 549 (0.6) | 14 (1.4) |  | 868 (0.4) | 8 (0.5) |  | 412 (0.4) | 34 (0.7) |  | 618 (0.5) | 9 (0.9) |
|  |  |  |  |  |  |  |  |  |  |  |  |  |  |  |  |
| Postoperative outcomes |  |  |  |  |  |  |  |  |  |  |  |  |  |  |  |
| Unscheduled intratracheal intubation | + | 1692 (5.2) | 7 (6.4) |  | 1175 (1.2) | 31 (3.2) |  | 1404 (0.7) | 33 (2.1) |  | 503 (0.5) | 88 (1.8) |  | 602 (0.5) | 14 (1.3) |
| Mechanical ventilation | + | 2186 (6.8) | 11 (10.0) |  | 1151 (1.2) | 49 (5.0) |  | 1315 (0.6) | 58 (3.7) |  | 546 (0.5) | 244 (5.0) |  | 610 (0.5) | 40 (3.8) |
| Blood transfusion | + | 2375 (7.3) | 5 (4.5) |  | 2904 (3.1) | 126 (12.9) |  | 3589 (1.8) | 117 (7.5) |  | 2122 (1.9) | 352 (7.2) |  | 1840 (1.6) | 63 (6.0) |
| ICU admission | + | 28,395 (87.9) | 96 (87.3) |  | 34,035 (35.8) | 494 (50.7) |  | 65,689 (32.1) | 700 (44.9) |  | 35,447 (32.5) | 2312 (47.2) |  | 38,035 (33.1) | 434 (41.4) |
| Re-operation | + | 1767 (5.5) | 9 (8.2) |  | 3007 (3.2) | 52 (5.3) |  | 4153 (2.0) | 70 (4.5) |  | 2915 (2.7) | 237 (4.8) |  | 7736 (6.7) | 83 (7.9) |
|  |  |  |  |  |  |  |  |  |  |  |  |  |  |  |  |
| Re-admission | + | 803 (2.5) | 4 (3.6) |  | 2764 (2.9) | 30 (3.1) |  | 4874 (2.4) | 54 (3.5) |  | 3345 (3.1) | 176 (3.6) |  | 3349 (2.9) | 43 (4.1) |
|  |  |  |  |  |  |  |  |  |  |  |  |  |  |  |  |
| Postoperative complications |  |  |  |  |  |  |  |  |  |  |  |  |  |  |  |
| Superficial incisional SSI | + | 2051 (6.3) | 10 (9.1) |  | 2298 (2.4) | 53 (5.4) |  | 3644 (1.8) | 89 (5.7) |  | 4562 (4.2) | 443 (9.0) |  | 3587 (3.1) | 86 (8.2) |
| Deep incisional SSI | + | 1016 (3.1) | 6 (5.5) |  | 971 (1.0) | 17 (1.7) |  | 1294 (0.6) | 24 (1.5) |  | 1143 (1.0) | 168 (3.4) |  | 1269 (1.1) | 33 (3.1) |
| Deep SSI | + | 2486 (7.7) | 7 (6.4) |  | 4755 (5.0) | 54 (5.5) |  | 5218 (2.6) | 53 (3.4) |  | 1793 (1.6) | 182 (3.7) |  | 6843 (6.0) | 81 (7.7) |
| Wound disruption | + | 466 (1.4) | 2 (1.8) |  | 519 (0.5) | 13 (1.3) |  | 811 (0.4) | 21 (1.3) |  | 680 (0.6) | 110 (2.2) |  | 602 (0.5) | 16 (1.5) |
| Anastomotic leakage | + | 3881 (12.0) | 8 (7.3) |  | 4183 (4.4) | 51 (5.2) |  | 4531 (2.2) | 53 (3.4) |  | 1651 (1.5) | 124 (2.5) |  | 10,247 (8.9) | 90 (8.6) |
| Pancreatic fistula | + | 165 (0.5) | 2 (1.8) |  | 4573 (4.8) | 37 (3.8) |  | 4837 (2.4) | 42 (2.7) |  | 211 (0.2) | 7 (0.1) |  | 82 (0.1) | 2 (0.2) |
| Pneumonia | + | 4094 (12.7) | 16 (14.5) |  | 3203 (3.4) | 47 (4.8) |  | 4160 (2.0) | 83 (5.3) |  | 1251 (1.1) | 215 (4.4) |  | 918 (0.8) | 22 (2.1) |
| Pulmonary embolism | + | 137 (0.4) | 0 (0.0) |  | 153 (0.2) | 5 (0.5) |  | 216 (0.1) | 5 (0.3) |  | 112 (0.1) | 9 (0.2) |  | 146 (0.1) | 0 (0.0) |
| Renal dysfunction | + | 482 (1.5) | 0 (0.0) |  | 860 (0.9) | 33 (3.4) |  | 1306 (0.6) | 43 (2.8) |  | 666 (0.6) | 156 (3.2) |  | 990 (0.9) | 28 (2.7) |
| Urinary infection | + | 159 (0.5) | 2 (1.8) |  | 534 (0.6) | 9 (0.9) |  | 956 (0.5) | 19 (1.2) |  | 585 (0.5) | 65 (1.3) |  | 1358 (1.2) | 9 (0.9) |
| CNS dysfunction | + | 106 (0.3) | 0 (0.0) |  | 243 (0.3) | 6 (0.6) |  | 413 (0.2) | 10 (0.6) |  | 219 (0.2) | 27 (0.6) |  | 185 (0.2) | 5 (0.5) |
| Prolonged disturbance of consciousness | + | 170 (0.5) | 0 (0.0) |  | 286 (0.3) | 12 (1.2) |  | 385 (0.2) | 15 (1.0) |  | 193 (0.2) | 74 (1.5) |  | 177 (0.2) | 7 (0.7) |
| Cardiac arrest | + | 196 (0.6) | 0 (0.0) |  | 373 (0.4) | 11 (1.1) |  | 494 (0.2) | 15 (1.0) |  | 210 (0.2) | 39 (0.8) |  | 221 (0.2) | 9 (0.9) |
| Acute myocardial infarction | + | 33 (0.1) | 0 (0.0) |  | 86 (0.1) | 2 (0.2) |  | 140 (0.1) | 3 (0.2) |  | 68 (0.1) | 9 (0.2) |  | 57 (0.0) | 3 (0.3) |
| Deep vein thrombosis | + | 209 (0.6) | 0 (0.0) |  | 248 (0.3) | 10 (1.0) |  | 335 (0.2) | 8 (0.5) |  | 259 (0.2) | 22 (0.4) |  | 253 (0.2) | 5 (0.5) |
| Sepsis | + | 1399 (4.3) | 10 (9.1) |  | 2019 (2.1) | 57 (5.8) |  | 2299 (1.1) | 72 (4.6) |  | 1039 (1.0) | 324 (6.6) |  | 2191 (1.9) | 67 (6.4) |
| CNS: central nervous system, ICU: intensive care unit, OR: odds ratio, SSI: surgical site infection | | | | | | | | | | | | | | | |
