# Supplementary material for: Emergency surgery for gastrointestinal cancer: A nationwide study in Japan based on the National Clinical Database
Source: Ann Gastroenterol Surg. 2020 Jun 21;4(5):549–61. doi: 10.1002/ags3.12353 (PMC7511565; doi:10.1002/ags3.12353)
Supplement: Supplementary file 2 — Table S2 [file AGS3-4-549-s002.docx]

| **Table S2** Risk factors for 30-day mortality: univariable analysis | | | | | | | | | | | | | | | | | | | | | | | | |
| --- | --- | --- | --- | --- | --- | --- | --- | --- | --- | --- | --- | --- | --- | --- | --- | --- | --- | --- | --- | --- | --- | --- | --- | --- |
|  |  | Total gastrectomy | | | | |  | Distal gastrectomy | | | | |  | Right hemicolectomy | | | | |  | Low anterior resection | | | | |
| Factor | Category | OR | 95% CI | | | P-value |  | OR | 95% CI | | | P-value |  | OR | 95% CI | | | P-value |  | OR | 95% CI | | | P-value |
| Preoperative factor |  |  |  |  |  |  |  |  |  |  |  |  |  |  |  |  |  |  |  |  |  |  |  |  |
| Age (years) | 70≤/<70 | 1.02 | 0.56 | - | 1.88 | 0.939 |  | 2.73 | 1.41 | - | 5.28 | 0.003 |  | 2.46 | 1.64 | - | 3.68 | <0.001 |  | 3.66 | 1.19 | - | 11.29 | 0.024 |
| Sex | Female/Male | 1.16 | 0.58 | - | 2.33 | 0.679 |  | 1.59 | 0.95 | - | 2.66 | 0.076 |  | 0.89 | 0.65 | - | 1.20 | 0.442 |  | 0.61 | 0.21 | - | 1.75 | 0.358 |
| Body mass index | 18.5≤, <25.0 | Reference | | | | |  | Reference | | | | |  | Reference | | | | |  | Reference | | | | |
|  | <18.5 | 1.80 | 0.95 | - | 3.42 | 0.073 |  | 1.71 | 1.00 | - | 2.95 | 0.052 |  | 1.61 | 1.16 | - | 2.24 | 0.005 |  | 1.02 | 0.28 | - | 3.74 | 0.976 |
|  | 25.0≤ | 0.55 | 0.17 | - | 1.86 | 0.339 |  | 0.46 | 0.16 | - | 1.30 | 0.142 |  | 0.77 | 0.45 | - | 1.32 | 0.345 |  | 1.80 | 0.56 | - | 5.82 | 0.325 |
| Diabetes mellitus | ± | 1.47 | 0.71 | - | 3.04 | 0.294 |  | 0.62 | 0.28 | - | 1.37 | 0.235 |  | 0.84 | 0.53 | - | 1.32 | 0.438 |  | 1.87 | 0.60 | - | 5.80 | 0.281 |
| Smoking | ± | 1.40 | 0.77 | - | 2.55 | 0.272 |  | 0.49 | 0.28 | - | 0.88 | 0.016 |  | 0.80 | 0.56 | - | 1.14 | 0.212 |  | 1.87 | 0.72 | - | 4.89 | 0.201 |
| Habitual drinking | ± | 1.17 | 0.61 | - | 2.23 | 0.634 |  | 0.84 | 0.46 | - | 1.54 | 0.570 |  | 0.80 | 0.52 | - | 1.22 | 0.298 |  | 1.94 | 0.73 | - | 5.16 | 0.182 |
| Dyspnea | ± | 5.87 | 2.64 | - | 13.05 | <0.001 |  | 3.27 | 1.43 | - | 7.52 | 0.005 |  | 4.01 | 2.45 | - | 6.58 | <0.001 |  | 0.00 | 0.00 | - | Inf | 0.991 |
| Dependence in ADL | ± | 4.14 | 2.22 | - | 7.73 | <0.001 |  | 3.44 | 2.04 | - | 5.81 | <0.001 |  | 4.28 | 3.15 | - | 5.83 | <0.001 |  | 4.61 | 1.67 | - | 12.72 | 0.003 |
| Mechanical ventilation | ± | 5.57 | 1.78 | - | 17.42 | 0.003 |  | 5.29 | 1.48 | - | 18.91 | 0.010 |  | 4.30 | 1.49 | - | 12.47 | 0.007 |  | 0.00 | 0.00 | - | Inf | 0.993 |
| COPD | ± | 1.68 | 0.50 | - | 5.65 | 0.405 |  | 0.41 | 0.06 | - | 2.99 | 0.377 |  | 1.67 | 0.81 | - | 3.47 | 0.168 |  | 5.59 | 1.21 | - | 25.83 | 0.027 |
| Pneumonia | ± | 8.23 | 2.11 | - | 32.15 | 0.002 |  | 2.83 | 0.83 | - | 9.60 | 0.096 |  | 4.05 | 2.05 | - | 7.99 | <0.001 |  | 19.50 | 3.74 | - | 101.77 | <0.001 |
| Ascites | ± | 3.30 | 1.61 | - | 6.76 | 0.001 |  | 3.17 | 1.75 | - | 5.74 | <0.001 |  | 1.73 | 1.19 | - | 2.51 | 0.004 |  | 4.36 | 1.38 | - | 13.72 | 0.012 |
| Esophageal varices | ± | 0.00 | 0.00 | - | Inf | 0.986 |  | 6.97 | 1.42 | - | 34.27 | 0.017 |  | 0.00 | 0.00 | - | Inf | 0.974 |  | 32.16 | 2.77 | - | 372.90 | 0.006 |
| Hypertension | ± | 1.18 | 0.64 | - | 2.18 | 0.588 |  | 0.96 | 0.57 | - | 1.63 | 0.890 |  | 1.67 | 1.23 | - | 2.27 | <0.001 |  | 2.64 | 1.01 | - | 6.91 | 0.048 |
| Congestive heart failure | ± | 10.77 | 1.92 | - | 60.41 | 0.007 |  | 0.00 | 0.00 | - | Inf | 0.980 |  | 3.61 | 1.70 | - | 7.66 | <0.001 |  | 0.00 | 0.00 | - | Inf | 0.991 |
| Angina pectoris | ± | 4.76 | 1.00 | - | 22.70 | 0.050 |  | 0.76 | 0.10 | - | 5.67 | 0.791 |  | 5.89 | 2.93 | - | 11.84 | <0.001 |  | 7.99 | 0.94 | - | 67.71 | 0.057 |
| Symptomatic PVD | ± | 0.00 | 0.00 | - | Inf | 0.985 |  | 0.00 | 0.00 | - | Inf | 0.982 |  | 11.97 | 3.07 | - | 46.69 | <0.001 |  | 0.00 | 0.00 | - | Inf | 0.993 |
| Acute renal failure | ± | 66.36 | 6.76 | - | 651.48 | <0.001 |  | 3.43 | 0.42 | - | 28.32 | 0.252 |  | 8.37 | 4.32 | - | 16.20 | <0.001 |  | 0.00 | 0.00 | - | Inf | 0.990 |
| Dialysis | ± | 2.62 | 0.32 | - | 21.41 | 0.369 |  | 3.20 | 0.94 | - | 10.96 | 0.064 |  | 5.23 | 2.30 | - | 11.90 | <0.001 |  | 21.42 | 2.11 | - | 217.15 | 0.010 |
| History of CVD | ± | 1.37 | 0.41 | - | 4.59 | 0.607 |  | 2.40 | 1.06 | - | 5.45 | 0.036 |  | 2.76 | 1.66 | - | 4.59 | <0.001 |  | 1.89 | 0.24 | - | 14.68 | 0.543 |
| Metastatic cancer | ± | 4.38 | 2.24 | - | 8.54 | <0.001 |  | 5.87 | 3.19 | - | 10.80 | <0.001 |  | 2.16 | 1.37 | - | 3.40 | <0.001 |  | 2.08 | 0.47 | - | 9.32 | 0.337 |
| Long-term steroid use | ± | 3.50 | 0.41 | - | 29.70 | 0.251 |  | 7.73 | 2.45 | - | 24.44 | <0.001 |  | 2.32 | 0.83 | - | 6.51 | 0.110 |  | 0.00 | 0.00 | - | Inf | 0.990 |
| Weight loss | ± | 4.33 | 2.28 | - | 8.25 | <0.001 |  | 1.69 | 0.84 | - | 3.40 | 0.138 |  | 1.26 | 0.74 | - | 2.12 | 0.396 |  | 2.28 | 0.51 | - | 10.20 | 0.282 |
| Blood clotting defects | ± | 5.46 | 2.62 | - | 11.39 | <0.001 |  | 2.50 | 1.24 | - | 5.05 | 0.011 |  | 3.16 | 2.06 | - | 4.85 | <0.001 |  | 1.51 | 0.20 | - | 11.66 | 0.693 |
| Chemotherapy | ± | 2.01 | 0.82 | - | 4.93 | 0.125 |  | 2.76 | 1.14 | - | 6.67 | 0.024 |  | 0.53 | 0.07 | - | 3.82 | 0.525 |  | 6.38 | 0.77 | - | 52.85 | 0.086 |
| Sepsis | ± | 3.99 | 1.68 | - | 9.48 | 0.002 |  | 5.14 | 2.80 | - | 9.40 | <0.001 |  | 5.02 | 3.60 | - | 7.00 | <0.001 |  | 3.47 | 1.11 | - | 10.88 | 0.033 |
| Blood transfusion | ± | 3.66 | 1.99 | - | 6.70 | <0.001 |  | 2.29 | 1.32 | - | 3.95 | 0.003 |  | 1.74 | 0.95 | - | 3.19 | 0.071 |  | 1.89 | 0.24 | - | 14.68 | 0.543 |
|  |  |  |  |  |  |  |  |  |  |  |  |  |  |  |  |  |  |  |  |  |  |  |  |  |
| Intraoperative factor |  |  |  |  |  |  |  |  |  |  |  |  |  |  |  |  |  |  |  |  |  |  |  |  |
| Endoscopy | ± | 0.80 | 0.19 | - | 3.40 | 0.764 |  | 0.08 | 0.01 | - | 0.61 | 0.014 |  | 0.40 | 0.19 | - | 0.81 | 0.011 |  | 0.00 | 0.00 | - | Inf | 0.988 |
| Diverting stoma | ± | - | - |  | - | - |  | - | - |  | - | - |  | - | - |  | - | - |  | 1.18 | 0.43 | - | 3.22 | 0.747 |
| Concurrent surgery | ± | 0.61 | 0.31 | - | 1.17 | 0.136 |  | 1.34 | 0.79 | - | 2.28 | 0.275 |  | 2.92 | 2.15 | - | 3.96 | <0.001 |  | 0.68 | 0.25 | - | 1.86 | 0.455 |
| ASA-PS | 1,2 | Reference | | | | |  | Reference | | | | |  | Reference | | | | |  | Reference | | | | |
|  | 3,4 | 6.64 | 3.02 | - | 14.62 | <0.001 |  | 5.55 | 3.10 | - | 9.93 | <0.001 |  | 5.01 | 3.56 | - | 7.06 | <0.001 |  | 6.72 | 2.31 | - | 19.53 | <0.001 |
|  | 5 | 24.00 | 7.54 | - | 76.39 | <0.001 |  | 7.69 | 2.10 | - | 28.15 | 0.002 |  | 16.21 | 8.47 | - | 31.02 | <0.001 |  | 17.11 | 1.81 | - | 161.58 | 0.013 |
| T | T3≤/≤T2 | 1.88 | 0.73 | - | 4.83 | 0.190 |  | 4.68 | 2.12 | - | 10.34 | <0.001 |  | 3.31 | 1.22 | - | 8.97 | 0.019 |  | 0.87 | 0.25 | - | 3.06 | 0.828 |
| N | N1≤/N0 | 2.31 | 1.02 | - | 5.25 | 0.044 |  | 2.45 | 1.36 | - | 4.42 | 0.003 |  | 1.04 | 0.77 | - | 1.42 | 0.783 |  | 1.29 | 0.49 | - | 3.41 | 0.609 |
| M | M1/M0 | 2.97 | 1.63 | - | 5.43 | <0.001 |  | 2.76 | 1.63 | - | 4.66 | <0.001 |  | 1.48 | 1.04 | - | 2.11 | 0.029 |  | 1.06 | 0.30 | - | 3.74 | 0.924 |
| Residual tumor | R1≤/R0 | 3.92 | 2.11 | - | 7.28 | <0.001 |  | 3.54 | 2.12 | - | 5.89 | <0.001 |  | 2.08 | 1.48 | - | 2.92 | <0.001 |  | 2.04 | 0.66 | - | 6.35 | 0.218 |
| ASA-PS: American Society of Anesthesiologists physical status, ADL: activities of daily living, COPD: chronic obstructive pulmonary disease, CVD: cerebrovascular disease, PVD: peripheral vascular disease | | | | | | | | | | | | | | | | | | | | | | | | |
