# Supplementary material for: Emergency surgery for gastrointestinal cancer: A nationwide study in Japan based on the National Clinical Database
Source: Ann Gastroenterol Surg. 2020 Jun 21;4(5):549–61. doi: 10.1002/ags3.12353 (PMC7511565; doi:10.1002/ags3.12353)
Supplement: Supplementary file 3 — Table S3 [file AGS3-4-549-s003.docx]

| **Table S3** Risk factors for overall postoperative complications: univariable analysis | | | | | | | | | | | | | | | | | | | | | | | | |
| --- | --- | --- | --- | --- | --- | --- | --- | --- | --- | --- | --- | --- | --- | --- | --- | --- | --- | --- | --- | --- | --- | --- | --- | --- |
|  |  | Total gastrectomy | | | | |  | Distal gastrectomy | | | | |  | Right hemicolectomy | | | | |  | Low anterior resection | | | | |
| Factor | Category | OR | 95% CI | | | P-value |  | OR | 95% CI | | | P-value |  | OR | 95% CI | | | P-value |  | OR | 95% CI | | | P-value |
| Preoperative factor |  |  |  |  |  |  |  |  |  |  |  |  |  |  |  |  |  |  |  |  |  |  |  |  |
| Age (years) | 70≤/<70 | 1.20 | 0.92 | - | 1.55 | 0.172 |  | 1.56 | 1.24 | - | 1.96 | <0.001 |  | 1.38 | 1.22 | - | 1.56 | <0.001 |  | 1.12 | 0.88 | - | 1.44 | 0.352 |
| Sex | Female/Male | 0.98 | 0.72 | - | 1.33 | 0.885 |  | 0.81 | 0.64 | - | 1.02 | 0.072 |  | 0.71 | 0.64 | - | 0.80 | <0.001 |  | 0.68 | 0.53 | - | 0.88 | 0.004 |
| Body mass index | 18.5≤, <25.0 | Reference | | | | |  | Reference | | | | |  | Reference | | | | |  | Reference | | | | |
|  | <18.5 | 1.57 | 1.15 | - | 2.14 | 0.004 |  | 1.21 | 0.94 | - | 1.56 | 0.146 |  | 1.20 | 1.04 | - | 1.37 | 0.011 |  | 1.26 | 0.91 | - | 1.73 | 0.160 |
|  | 25.0≤ | 1.09 | 0.74 | - | 1.60 | 0.674 |  | 1.26 | 0.93 | - | 1.70 | 0.140 |  | 1.13 | 0.95 | - | 1.34 | 0.170 |  | 1.49 | 1.05 | - | 2.12 | 0.026 |
| Diabetes mellitus | ± | 0.96 | 0.68 | - | 1.35 | 0.807 |  | 1.36 | 1.03 | - | 1.79 | 0.029 |  | 1.21 | 1.03 | - | 1.42 | 0.021 |  | 1.34 | 0.95 | - | 1.90 | 0.099 |
| Smoking | ± | 1.03 | 0.79 | - | 1.33 | 0.846 |  | 1.07 | 0.86 | - | 1.33 | 0.529 |  | 1.24 | 1.09 | - | 1.40 | 0.001 |  | 1.36 | 1.05 | - | 1.75 | 0.019 |
| Habitual drinking | ± | 1.00 | 0.75 | - | 1.33 | 0.986 |  | 0.75 | 0.58 | - | 0.96 | 0.021 |  | 1.10 | 0.94 | - | 1.27 | 0.225 |  | 1.28 | 0.97 | - | 1.69 | 0.079 |
| Dyspnea | ± | 1.71 | 0.95 | - | 3.07 | 0.075 |  | 2.55 | 1.53 | - | 4.25 | <0.001 |  | 2.59 | 1.89 | - | 3.53 | <0.001 |  | 1.81 | 0.80 | - | 4.07 | 0.154 |
| Dependence in ADL | ± | 1.59 | 1.12 | - | 2.27 | 0.010 |  | 1.94 | 1.48 | - | 2.54 | <0.001 |  | 2.00 | 1.75 | - | 2.29 | <0.001 |  | 2.05 | 1.39 | - | 3.03 | <0.001 |
| Mechanical ventilation | ± | 1.81 | 0.74 | - | 4.41 | 0.192 |  | 1.80 | 0.69 | - | 4.70 | 0.228 |  | 2.90 | 1.38 | - | 6.10 | 0.005 |  | 3.02 | 0.27 | - | 33.46 | 0.367 |
| COPD | ± | 1.28 | 0.68 | - | 2.39 | 0.447 |  | 1.55 | 0.91 | - | 2.64 | 0.104 |  | 2.41 | 1.71 | - | 3.38 | <0.001 |  | 3.50 | 1.51 | - | 8.12 | 0.004 |
| Pneumonia | ± | 2.59 | 0.75 | - | 8.90 | 0.132 |  | 2.92 | 1.38 | - | 6.16 | 0.005 |  | 2.22 | 1.42 | - | 3.46 | <0.001 |  | 5.35 | 1.11 | - | 25.87 | 0.037 |
| Ascites | ± | 1.39 | 0.91 | - | 2.12 | 0.133 |  | 1.73 | 1.25 | - | 2.41 | 0.001 |  | 1.68 | 1.43 | - | 1.98 | <0.001 |  | 1.46 | 0.91 | - | 2.37 | 0.119 |
| Esophageal varices | ± | 0.49 | 0.05 | - | 4.69 | 0.533 |  | 1.01 | 0.25 | - | 4.05 | 0.991 |  | 3.34 | 1.14 | - | 9.80 | 0.028 |  | 3.02 | 0.27 | - | 33.46 | 0.367 |
| Hypertension | ± | 1.19 | 0.92 | - | 1.56 | 0.192 |  | 1.25 | 1.00 | - | 1.55 | 0.046 |  | 1.32 | 1.17 | - | 1.49 | <0.001 |  | 1.13 | 0.87 | - | 1.48 | 0.368 |
| Congestive heart failure | ± | 2.94 | 0.54 | - | 16.15 | 0.214 |  | 3.33 | 1.37 | - | 8.10 | 0.008 |  | 2.59 | 1.61 | - | 4.19 | <0.001 |  | 3.56 | 0.92 | - | 13.84 | 0.067 |
| Angina pectoris | ± | 3.96 | 1.04 | - | 15.02 | 0.043 |  | 2.05 | 1.02 | - | 4.13 | 0.045 |  | 1.99 | 1.19 | - | 3.33 | 0.009 |  | 5.35 | 1.11 | - | 25.87 | 0.037 |
| Symptomatic PVD | ± | 0.97 | 0.16 | - | 5.86 | 0.978 |  | 0.81 | 0.16 | - | 4.17 | 0.796 |  | 15.06 | 1.91 | - | 118.93 | 0.010 |  | 3.02 | 0.27 | - | 33.46 | 0.367 |
| Acute renal failure | ± | 4.41 | 0.46 | - | 42.57 | 0.199 |  | 6.11 | 1.23 | - | 30.36 | 0.027 |  | 3.11 | 1.77 | - | 5.45 | <0.001 |  | 7.62 | 0.89 | - | 65.41 | 0.064 |
| Dialysis | ± | 2.95 | 0.73 | - | 11.88 | 0.127 |  | 2.39 | 1.10 | - | 5.20 | 0.028 |  | 2.30 | 1.27 | - | 4.16 | 0.006 |  | 4.55 | 0.47 | - | 43.86 | 0.190 |
| History of CVD | ± | 3.20 | 1.74 | - | 5.90 | <0.001 |  | 2.16 | 1.38 | - | 3.38 | <0.001 |  | 1.85 | 1.40 | - | 2.44 | <0.001 |  | 1.73 | 0.87 | - | 3.43 | 0.118 |
| Metastatic cancer | ± | 1.37 | 0.91 | - | 2.07 | 0.137 |  | 1.94 | 1.29 | - | 2.93 | 0.002 |  | 1.32 | 1.06 | - | 1.65 | 0.014 |  | 1.11 | 0.66 | - | 1.85 | 0.698 |
| Long-term steroid use | ± | 3.69 | 0.71 | - | 19.11 | 0.120 |  | 2.92 | 1.10 | - | 7.71 | 0.031 |  | 1.22 | 0.70 | - | 2.13 | 0.474 |  | 3.03 | 0.55 | - | 16.64 | 0.201 |
| Weight loss | ± | 1.46 | 1.00 | - | 2.14 | 0.052 |  | 1.16 | 0.83 | - | 1.64 | 0.382 |  | 1.45 | 1.17 | - | 1.80 | <0.001 |  | 3.70 | 2.09 | - | 6.53 | <0.001 |
| Blood clotting defects | ± | 2.19 | 1.30 | - | 3.68 | 0.003 |  | 2.10 | 1.43 | - | 3.08 | <0.001 |  | 1.72 | 1.36 | - | 2.18 | <0.001 |  | 3.16 | 1.64 | - | 6.08 | <0.001 |
| Chemotherapy | ± | 0.93 | 0.57 | - | 1.51 | 0.757 |  | 1.06 | 0.62 | - | 1.82 | 0.831 |  | 1.28 | 0.74 | - | 2.21 | 0.375 |  | 1.26 | 0.38 | - | 4.15 | 0.705 |
| Sepsis | ± | 2.33 | 1.29 | - | 4.22 | 0.005 |  | 2.18 | 1.47 | - | 3.22 | <0.001 |  | 3.00 | 2.47 | - | 3.64 | <0.001 |  | 2.89 | 1.84 | - | 4.55 | <0.001 |
| Blood transfusion | ± | 1.55 | 1.17 | - | 2.07 | 0.003 |  | 1.82 | 1.40 | - | 2.38 | <0.001 |  | 1.32 | 1.00 | - | 1.75 | 0.051 |  | 1.53 | 0.77 | - | 3.03 | 0.224 |
|  |  |  |  |  |  |  |  |  |  |  |  |  |  |  |  |  |  |  |  |  |  |  |  |  |
| Intraoperative factor |  |  |  |  |  |  |  |  |  |  |  |  |  |  |  |  |  |  |  |  |  |  |  |  |
| Endoscopy | ± | 0.68 | 0.37 | - | 1.22 | 0.195 |  | 0.51 | 0.37 | - | 0.71 | <0.001 |  | 0.43 | 0.35 | - | 0.54 | <0.001 |  | 0.78 | 0.58 | - | 1.04 | 0.088 |
| Diverting stoma | ± | - | - |  | - | - |  | - | - |  | - | - |  | - | - |  | - | - |  | 1.43 | 1.10 | - | 1.86 | 0.008 |
| Concurrent surgery | ± | 1.16 | 0.89 | - | 1.50 | 0.273 |  | 1.43 | 1.14 | - | 1.80 | 0.002 |  | 2.32 | 2.03 | - | 2.65 | <0.001 |  | 1.27 | 0.99 | - | 1.63 | 0.058 |
| ASA-PS | 1,2 | Reference | | | | |  | Reference | | | | |  | Reference | | | | |  | Reference | | | | |
|  | 3,4 | 1.55 | 1.19 | - | 2.02 | 0.001 |  | 1.90 | 1.52 | - | 2.37 | <0.001 |  | 2.26 | 2.00 | - | 2.56 | <0.001 |  | 2.32 | 1.74 | - | 3.08 | <0.001 |
|  | 5 | 4.36 | 1.78 | - | 10.68 | 0.001 |  | 9.27 | 3.70 | - | 23.22 | <0.001 |  | 4.99 | 3.00 | - | 8.30 | <0.001 |  | 7.65 | 1.61 | - | 36.30 | 0.010 |
| T | T3≤/≤T2 | 1.49 | 1.06 | - | 2.10 | 0.020 |  | 1.67 | 1.33 | - | 2.10 | <0.001 |  | 1.63 | 1.28 | - | 2.08 | <0.001 |  | 1.76 | 1.22 | - | 2.52 | 0.002 |
| N | N1≤/N0 | 1.25 | 0.94 | - | 1.66 | 0.123 |  | 1.51 | 1.21 | - | 1.87 | <0.001 |  | 0.96 | 0.85 | - | 1.08 | 0.462 |  | 1.13 | 0.88 | - | 1.45 | 0.322 |
| M | M1/M0 | 1.10 | 0.82 | - | 1.46 | 0.522 |  | 1.40 | 1.08 | - | 1.82 | 0.011 |  | 1.09 | 0.94 | - | 1.27 | 0.230 |  | 1.15 | 0.82 | - | 1.59 | 0.418 |
| Residual tumor | R1≤/R0 | 1.12 | 0.85 | - | 1.48 | 0.403 |  | 1.41 | 1.10 | - | 1.81 | 0.007 |  | 1.29 | 1.11 | - | 1.51 | <0.001 |  | 1.58 | 1.11 | - | 2.26 | 0.012 |
| ASA-PS: American Society of Anesthesiologists physical status, ADL: activities of daily living, COPD: chronic obstructive pulmonary disease, CVD: cerebrovascular disease, PVD: peripheral vascular disease | | | | | | | | | | | | | | | | | | | | | | | | |
