# Supplementary material for: Emergency surgery for gastrointestinal cancer: A nationwide study in Japan based on the National Clinical Database
Source: Ann Gastroenterol Surg. 2020 Jun 21;4(5):549–61. doi: 10.1002/ags3.12353 (PMC7511565; doi:10.1002/ags3.12353)
Supplement: Supplementary file 4 — Supplementary Material [file AGS3-4-549-s004.docx]

**Appendix S1** Details of secondary outcomes

Intraoperative outcomes: anesthesia time, operating time, estimated blood loss, blood transfusion, and incidence of intraoperative adverse events.

Postoperative outcomes: length of hospital stay, incidence of repeat surgery within 30 days after the primary surgery, re-admission within 30 days after the primary surgery, and admission to the intensive care unit.

Postoperative complications: superficial incisional surgical site infection (SSI), deep incisional SSI, deep SSI, wound disruption, anastomotic leakage, pancreatic fistula, pneumonia, unscheduled intratracheal intubation, pulmonary embolism, mechanical ventilation, renal dysfunction, urinary infection, central nervous system dysfunction, prolonged disturbance of consciousness (>24 h), cardiac arrest, acute myocardial infarction, postoperative blood transfusion, deep vein thrombosis, and sepsis.
