# Supplementary material for: Emergency surgery for gastrointestinal cancer: A nationwide study in Japan based on the National Clinical Database
Source: Ann Gastroenterol Surg. 2020 Jun 21;4(5):549–61. doi: 10.1002/ags3.12353 (PMC7511565; doi:10.1002/ags3.12353)
Supplement: Supplementary file 5 — Supplementary Material [file AGS3-4-549-s005.docx]

**Appendix S2** Details of risk factors for the 30-day mortality and incidence of overall postoperative complications

Preoperative factors: age, sex, body mass index, diabetes mellitus, smoking, habitual drinking, dyspnea, dependence in activities of daily living, mechanical ventilation, chronic obstructive pulmonary disease, pneumonia, ascites, esophageal varices, hypertension, congestive heart failure, angina pectoris, symptomatic peripheral vascular disease, acute renal failure, dialysis, history of cerebrovascular disease, metastatic cancer, long-term steroid use, weight loss, blood clotting defects, chemotherapy, sepsis, and blood transfusion.

Intraoperative factors: endoscopy, diverting stoma, concurrent surgery, American Society of Anesthesiologists physical status, T staging, N staging, M staging, and residual tumor.
