# Supplementary material for: Emergency surgery for gastrointestinal cancer: A nationwide study in Japan based on the National Clinical Database
Source: Ann Gastroenterol Surg. 2020 Jun 21;4(5):549–61. doi: 10.1002/ags3.12353 (PMC7511565; doi:10.1002/ags3.12353)
Supplement: Supplementary file 6 — Supplementary Material [file AGS3-4-549-s006.docx]

**Appendix S3** Details of potentially critical factors for 30-day mortality

Total gastrectomy group: dyspnea, dependence in activities of daily living (ADL), mechanical ventilation, pneumonia, ascites, congestive heart failure, acute renal failure, metastatic cancer in another organ, weight loss, blood clotting defects, sepsis, and high American Society of Anesthesiologists physical status (ASA-PS).

Distal gastrectomy group: dyspnea, dependence in ADL, mechanical ventilation, ascites, esophageal varices, metastatic cancer in another organ, long-term steroid use, sepsis, high ASA-PS, and advanced T stage.

Right hemicolectomy group: dyspnea, dependence in ADL, mechanical ventilation, pneumonia, congestive heart failure, angina pectoris, symptomatic peripheral vascular disease, acute renal failure, dialysis, blood clotting defects, sepsis, high ASA-PS, and advanced T stage.

Low anterior resection group: being elderly, dependence in ADL, chronic obstructive pulmonary disease, pneumonia, ascites, esophageal varices, dialysis, preoperative chemotherapy, sepsis, and high ASA-PS.
